# Temporal Dynamics of the Nasopharyngeal Microbiome and its Relationship with Childhood Asthma Exacerbation

**DOI:** 10.1128/spectrum.00129-22

**Published:** 2022-05-12

**Authors:** Jinpao Hou, Yuping Song, Agnes Sze Yin Leung, Man Fung Tang, Mai Shi, Evy Yiwei Wang, Joseph Gar Shun Tsun, Renee Wan Yi Chan, Gary Wing Kin Wong, Stephen Kwok-Wing Tsui, Ting Fan Leung

**Affiliations:** a School of Biomedical Sciences, The Chinese University of Hong Kong, Hong Kong, China; b Hong Kong Bioinformatics Center, The Chinese University of Hong Kong, Hong Kong, China; c Department of Pediatrics, The Chinese University of Hong Kong, Hong Kong, China; d Department of Pediatrics, Prince of Wales Hospital, Hong Kong, China; e Chinese University of Hong Kong-University Medical Center Utrecht Joint Research Laboratory of Respiratory Virus and Immunobiology, The Chinese University of Hong Kong, Hong Kong, China; f Hong Kong Hub of Pediatric Excellence, The Chinese University of Hong Kong, Hong Kong, China; g Center for Microbial Genomics and Proteomics, The Chinese University of Hong Kong, Hong Kong, China; Wright State University

**Keywords:** asthma exacerbation, dynamics, longitudinal sampling, microbiome profile group, nasopharyngeal microbiome

## Abstract

Despite distinct nasopharyngeal microbiome (NPM) profiles between asthmatics and healthy subjects, little is known about the NPM dynamics and its relation to childhood asthma exacerbation (AE). We investigated NPM changes by longitudinally collecting 135 flocked nasopharyngeal swabs (FNPSs) from 33 school-age asthmatic children at six time points (2 to 4-week intervals) from September to December 2017 in Hong Kong. Subjects were categorized into AE and stable asthma (AS) groups according to whether they experienced any exacerbation during follow-up. One-off FNPSs from nine nonasthmatic children were included as controls. Microbiota profiles were analyzed using 16S rRNA gene sequencing. All 144 NPMs were classified into six microbiome profile groups (MPGs), each dominated by *Moraxella*, *Corynebacterium 1*, *Dolosigranulum*, Staphylococcus, Streptococcus, or *Anoxybacillus*. The microbial diversity and compositions of NPM in exacerbation samples were different from both baseline samples and those from healthy controls. *Moraxella* and *Dolosigranulum*-dominated NPM exhibited high temporal stability revealed by MPG transition analysis. NPM diversity decreased whereas microbial composition remained similar over time. The relative abundances of *Moraxella* increased while *Corynebacterium 1*, *Anoxybacillus*, and Pseudomonas decreased longitudinally. However, these temporal patterns did not differ between AE and AS groups, suggesting that short-term dynamic patterns were not sufficient to predict AE occurrence. Asthmatic NPM underwent *Moraxella* expansion during AE and presented a high microbiome resilience (recovery potential) after AE resolution. Microbial pathways involved in methane, ketone bodies, and vitamin B3 metabolisms were enhanced during AE and primarily contributed by *Moraxella*.

**IMPORTANCE** Evidence on the dynamic changes of NPM in asthmatic patients remains limited. Here, we present that asthmatic NPMs deviating from a healthy status still showed resilience after disturbance. Our data imply from a longitudinal perspective that *Moraxella* increase is closely related to AE occurrence. The finding of functional dysbiosis (imbalance) during AE offers a plausible explanation for the known association between nasopharyngeal *Moraxella* expansion and increased AE risk. This work serves as a basis for future long-term prospective studies leveraging multiomics approaches to elucidate the temporal association between NPM and pediatric AE.

## INTRODUCTION

Asthma is a chronic respiratory disorder characterized by recurrent, reversible constriction of the lower airways (1). Despite the standardized management, asthma exacerbation (AE) continues to occur, which leads to compromised health-related quality of life for patients and families as well as a considerable health care burden to society (2, 3). Although childhood AE has long been attributed to respiratory viral infection, emerging evidence indicates that airway bacteria also play a part in modulating this event (4–7). Ever since the conceptual misunderstanding of “sterile airway” was overthrown by studies based on advanced culture-independent microbial detection technologies, the relationship between airway microbiome and respiratory disorders has been widely investigated (8, 9). These studies revealed that airway microbial dysbiosis was found in patients with different asthma phenotypes (6, 10–12). However, the relationship between the airway microbiome and AE remains poorly understood. Given nasopharynx is a reservoir for upper respiratory tract pathogens involved in asthma pathophysiology, we previously reported that preschool children hospitalized for wheezing disorder harbored more abundant Proteobacteria and less abundant *Dolosigranulum* (Firmicutes) in the nasopharynx than community control (13). This finding is consistent with an adult study in which nasal Proteobacteria and Bacteroidetes were more enriched in patients with AE than in healthy individuals (14). This study also reported higher *Prevotella* (Bacteroidetes), *Alkanindiges* (Proteobacteria), and *Gardnerella* (Actinobacteria) but lower *Dialister* (Firmicutes) in the nasal microbiome during AE. Nevertheless, these cross-sectional studies provided a snapshot of the airway microbiome at only one time point.

There were few longitudinal studies on the temporal dynamics of the nasopharyngeal microbiome (NPM) in asthmatic children. Zhou et al. reported that nasal microbiome transition from *Corynebacterium and Dolosigranulum* cluster at baseline to *Moraxella* cluster at the time of loss of asthma control had the highest risk for AE in school-age asthmatics (15). The relatively long sampling interval (1 year) in this study, however, had limited its ability to capture transient changes in NPM. Through biweekly sampling from 478 asthmatic children in the PROSE study, McCauley et al. (16) demonstrated that AE was associated with the *Moraxella*-dominated nasal microbiome. These studies longitudinally interrogated the relationship between NPM and childhood AE among American cohorts. Despite the similarity of a healthy microbiome in the upper respiratory tract across geographically diverse populations (17), it remains unclear whether different patterns exist regarding temporal dynamics of NPM diversity and composition as well as its relationship with AE in Asian children compared to their western counterparts.

In this prospective study, we performed 16S rRNA gene profiling of NPM to assess its temporal dynamics among Hong Kong schoolchildren with asthma. We aimed to address the following knowledge gaps: (i) longitudinal changes in NPM diversity and composition and their association with AE, and (ii) taxonomical and functional changes of NPM, especially for the dominant genera, at the time of AE.

## RESULTS

### Study population and clinical outcomes.

**FIG 1:**
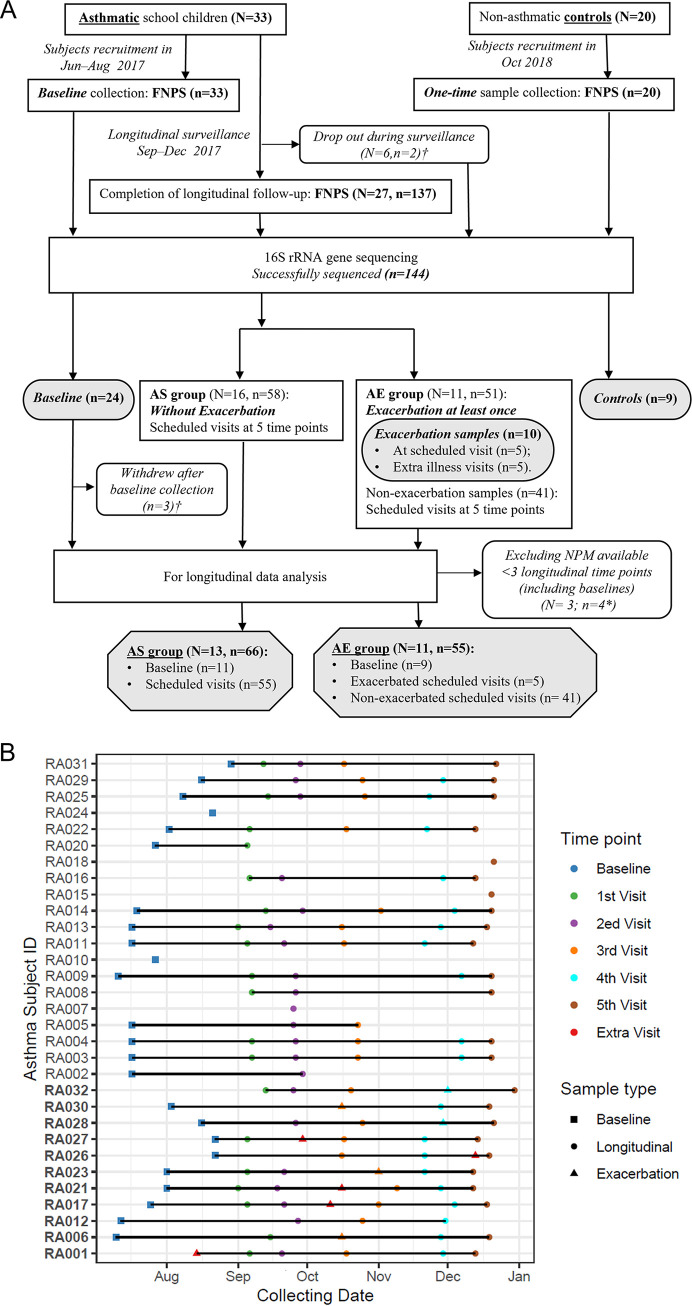
Study design and sample collection. (A) Flowchart of subject selection. N, number of subjects; n, number of samples. Samples in elliptic boxes colored in gray were used for cross-sectional comparisons; samples in polygon boxes colored in gray were included for longitudinal NPM analyses. † Four subjects withdrew after baseline sample collection, and 2 subjects withdrew after the first visit. Their samples were sequenced but were excluded from longitudinal analyses. *, these 4 samples were from the AS group, including 1 baseline and 3 longitudinal samples collected at scheduled visits. FNPS flocked nasopharyngeal swab; AE, asthma exacerbation; AS, stable asthma. (B) Horizontal lines show the collection timing of 135 longitudinal samples that were successfully sequenced in all asthmatic subjects. Dots refer to samples colored by time point. Triangles indicate exacerbation samples and circles longitudinal nonexacerbation samples. The exacerbation sample from subject number RA012 and the baseline samples from number RA019 and number RA033 were not successfully sequenced. The latter two subjects were not shown as they withdrew before the follow-up period.

A total of 33 asthmatics and 20 nonasthmatic controls were recruited in this study (Fig. 1A). Flocked nasopharyngeal swabs (FNPSs, *n* = 172) from the asthmatics were longitudinally collected from baseline until the end of surveillance (i.e., six time points), whereas only one-off cross-sectional FNPSs were obtained from controls at recruitment. Twenty-seven (81.8%) asthmatic children completed the follow-up period, during which a total of 12 episodes of exacerbation occurred in 11 (40.7%) subjects (AE group) (Fig. 1A). Six of these 12 exacerbation events occurred within 2 days of the scheduled visits and thus no extra visits were arranged; one of these 6 exacerbation samples was excluded due to amoxicillin-clavulanate treatment for otitis media before sample collection. The exacerbation samples from the other six episodes were collected at extra illness visits (Fig. 1A). The remaining subjects who did not experience any exacerbation events during the surveillance period were considered the stable asthma (AS) group. Among the 172 asthmatic FNPSs obtained, human rhinovirus (HRV) was detected in 41 (23.8%) samples, including 26.8% HRV-A (*n* = 11), 34.1% HRV-B (*n* = 14), and 36.6% HRV-C (*n* = 15). One sample with inconclusive sequences did not map to any HRV subtype. HRV was also detected in three control samples, for which no genotyping was conducted due to weak signals.

### Six major genera in NPM.

**FIG 2:**
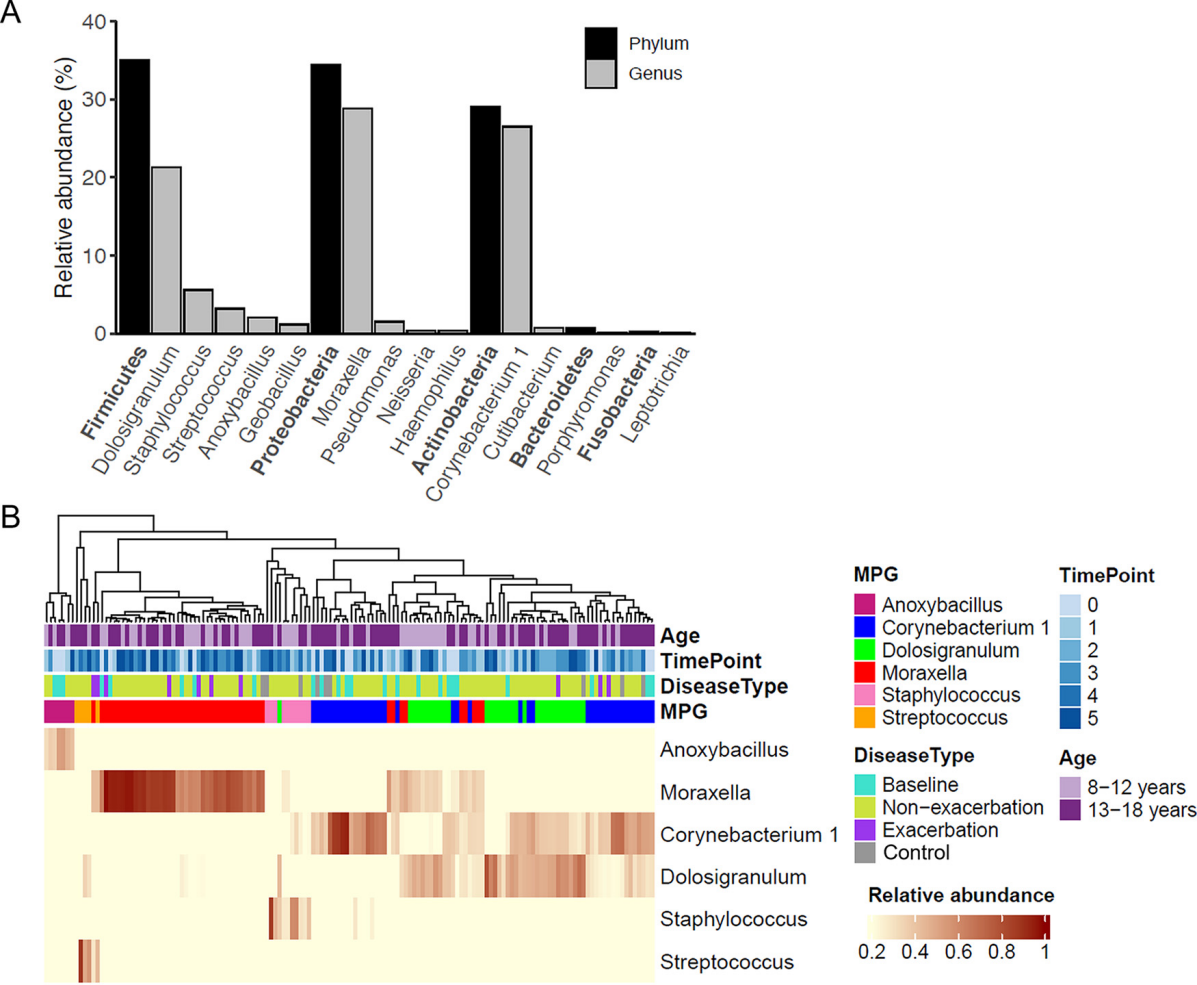
The major phyla and genera of the nasopharyngeal microbiome in all 144 samples. (A) Bar plots showing the average relative abundances of the major phyla and their corresponding genera across all samples. (B) Complete linkage hierarchical clustering based on Bray-Curtis distances assigned samples into six MPGs according to the most abundant genus in each sample. MPGs were color-coded as to their dominant genera. Time points, age, and sample type were also color-coded as indicated. Timepoint 0 refers to baseline. Control, nonasthmatic controls; Baseline, asthmatic samples collected at baseline; Nonexacerbation, longitudinal samples collected when AE episodes did not occur; Exacerbation, all exacerbated samples collected at both scheduled and extra visits.

NPM profiles were successfully recovered from 144 samples (135 asthmatics and 9 controls, Fig. 1A). An average of 5.3 samples per patient were available for downstream analyses (Fig. 1B). We obtained over 9.29 million high-quality sequences (median 75,365, interquartile range [IQR] 46,501-79,697) after denoising using DADA2. These sequences corresponded to 5,209 ASVs with 10 to 476 ASVs (mean 99) per sample after excluding the rare (total frequency <2) ones, which were taxonomically assigned to 30 phyla and 736 genera. The major phyla of NPM in schoolchildren were Firmicutes (35%), Proteobacteria (34.5%), Actinobacteria (29%), Bacteroidetes (0.7%) and Fusobacteria (0.3%) (Fig. 2A). All samples were categorized into six MPGs, with each dominated by one of the following genera: *Moraxella* (34%, 49 samples), *Corynebacterium 1* (28.5%, 41 samples), *Dolosigranulum* (22.2%, 32 samples), Staphylococcus (6.9%, 10 samples), Streptococcus (3.5%, 5 samples) and *Anoxybacillus* (4.9%, 7 samples). Additionally, hierarchical clustering of samples based on relative abundances of these six genera revealed that the *Moraxella* MPG was mainly from longitudinal asthmatic samples, whereas baseline and healthy control samples primarily belonged to *Dolosigranulum* or *Corynebacterium 1* MPGs (Fig. 2B). Of note, a high abundance of *Corynebacterium 1* was also found in some nonexacerbated samples assigned to the *Dolosigranulum*-dominated MPG.

### Cross-sectional comparison of NPM diversity and composition between asthmatics and controls.

Asthmatic NPM composition (beta-diversity) at baseline was significantly associated with age (permutational multivariate analysis of variance [PERMANOVA] test on Bray-Curtis distance, *P* = 0.014), concomitant atopy and exposure to house dust mites (HDM, *P* = 0.016), whereas it did not correlate with AE occurrence (R^2^ = 0.006, *P* = 0.993) and HRV infection (R^2^ = 0.061, *P* = 0.306; Table 1). Notably, NPM alpha diversity represented by the Shannon diversity index (SDI) at baseline was not correlated with age (Spearman’s ρ = 0.04, *P* = 0.842).

**TABLE 1:** Relationship between clinical factors and NPM composition at baseline

Variable	R^2^	*P* value
Asthma group^*a*^	0.006	0.993
Gender	0.036	0.636
Age	0.135	**0.014**
Human rhinovirus infection	0.061	0.306
Inhaled corticosteroid treatment	0.026	0.828
Underweight	0.072	0.190
Pet keeping	0.064	0.241
Smoking exposure	0.022	0.920
Shared bedroom	0.021	0.928
Presence of siblings	0.043	0.544
Asthma control	0.036	0.657
Concomitant HDM atopy and exposure	0.132	**0.016**

a Asthma exacerbation (AE) versus stable asthma (AS). *P* value and R^2^ were calculated based on Bray-Curtis distance with Adonis PERMANOVA (1000 permutations) test using vegan R package; significant *P* values (<0.05) were shown in bold.

**FIG 3:**
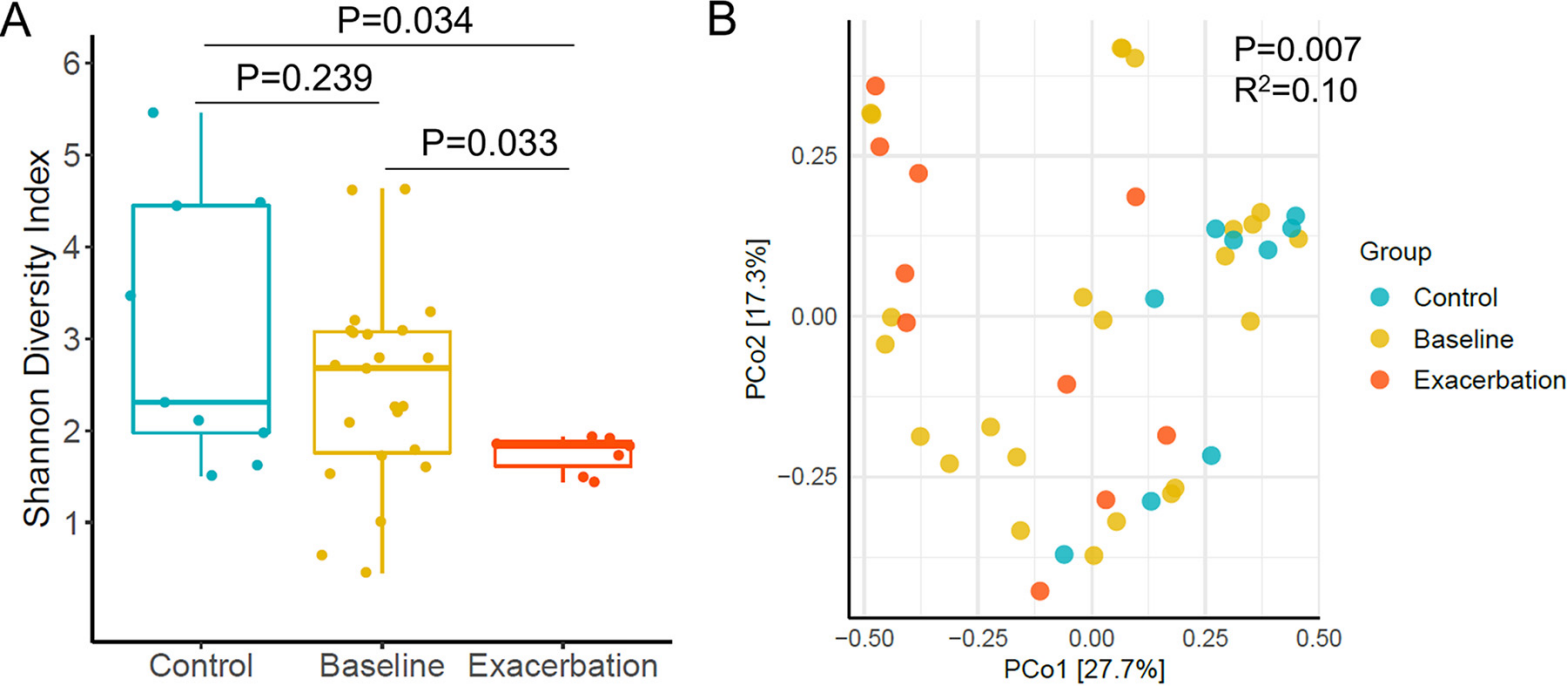
Distinct alpha and beta diversity of NPM between asthmatic and healthy subjects. (A) Shannon diversity index in controls, asthmatic baseline, and exacerbated samples. Kruskal-Wallis test was conducted followed by Dunn’s *post hoc* test with FDR correction. Boxes represent the median, lower and upper quartiles, respectively. The ends of the whiskers indicate the minimum and maximum of the data after the removal of outliers. (B) Principal coordinate analysis (PCoA) plot based on Bray-Curtis distances. Points refer to samples that were colored by the group. Control, nonasthmatic controls; Baseline, asthmatic samples collected at baseline. Exacerbation, all exacerbated samples were collected at both scheduled and extra visits.

To evaluate the NPM variations at different clinical asthma statuses relative to a healthy NPM, we compared the alpha and beta diversity among asthmatic baseline (*n* = 24), exacerbation (*n* = 10), and healthy control (*n* = 9) samples (Fig. 1A). Overall, SDI was lower in exacerbation samples than that at baseline (geometric mean 1.71 versus 2.18, *P*_adj_ = 0.033) and controls (geometric mean 2.75, *P*_adj_ = 0.034; Fig. 3A). The microbial composition of the nasopharynx was also different across these three sample categories after adjustment for age (R^2^=0.1, *P*_adj_ = 0.007). This was also confirmed by a clear separation of control samples from both exacerbation and baseline samples (pairwise adonis, *P*_adj_ = 0.003 and *P*_adj_ = 0.044, respectively; Fig. 3B) as shown in the principal coordinate analysis (PCoA) plot based on Bray-Curtis distance. To further understand the NPM shifts during exacerbation within the same asthmatic patient, we performed a pairwise comparison of microbial diversity in eight pairs of baseline-exacerbation samples. A lower alpha diversity (Wilcoxon signed-rank test, *P* = 0.031; Fig. S2A) was observed in exacerbation samples than in the paired baseline group. Procrustes analysis of Bray-Curtis distances also revealed an insignificant correlation (Mantel test, *P* = 0.116, R^2^ = 0.073; Fig. S2B) between paired samples, suggesting a remarkable variation in NPM composition during AE. Taken together, these observations suggested aberrant microbial diversity and composition of the nasopharynx at AE compared to both baseline and healthy controls.

### Temporal stability of *Moraxella-*and *Dolosigranulum*-dominated NPM.

**FIG 4:**
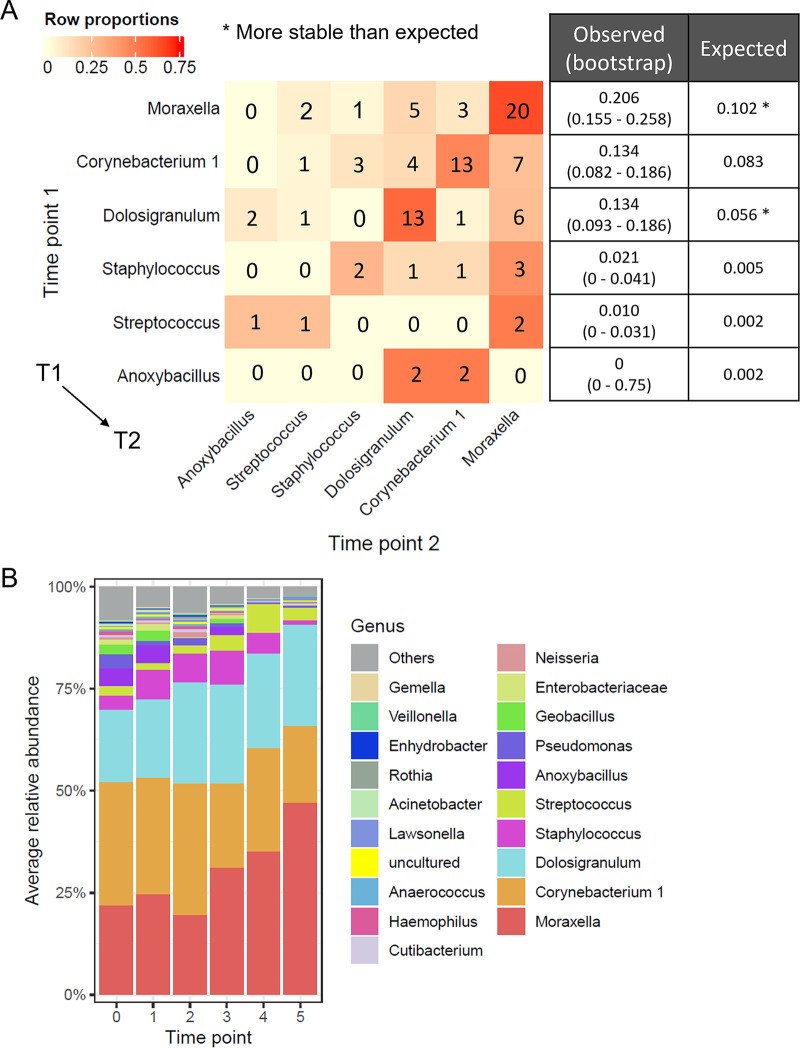
Stability and temporal dynamics of NPM in asthmatic children (*n* = 121). (A) MPG transition matrix between consecutive samples from the same subject for all patients. T1, 1st-time point; T2, next time point. Cell numbers refer to the number of cases in which the corresponding transition from T1 to T2 was observed. Cells are color-coded to indicate row proportions as shown in the legend. The numbers of stable transitions for each MPG were shown as the second diagonal of the matrix, meaning that the same MPG was observed at T1 and T2. The table in the right panel shows observed and expected frequencies of stable transitions for the corresponding MPG with their names labeled on the left side of the matrix. (B) Stacked bar plots showing the relative abundances of the top 20 most abundant genera by time point. Genera were ordered by their average relative abundances and those beyond the top 20 were collapsed as ‘Others’. Time point 0 refers to baseline.

We next investigated the MPG transitions between consecutive time points in 121 asthmatic longitudinal samples, regardless of whether they were from AE or AS subjects. Stable MPG transition was defined as the same MPG being observed in samples at the subsequent time point from the same subject. For all asthmatic subjects, a pairwise assessment of transitions between the six MPGs was performed to determine the frequency of stable transition for each MPG. For the *Moraxella* MPG and *Dolosigranulum* MPG, the observed frequency of stable transition was significantly higher than the expected frequency (Fig. 4A), suggesting temporal stability of NPMs dominated by each of these two genera. When stable colonization of a given genus was defined as >50% of a patient’s longitudinal samples belonging to the MPG dominated by that genus, we observed that 30.8% (4/13) of AS patients had NPM stably colonized by a *Corynebacterium 1* whereas none of AE patients’ NPM was stably colonized by this genus (Fisher’s exact test, *P* = 0.098). Such observation implied that *Corynebacterium 1* might protect against AE. Furthermore, we assessed the temporal trends in relative abundances of these MPG genera using linear mixed-effect (LME) models. The results indicated that *Moraxella* steadily increased (*P* = 0.001), whereas *Corynebacterium 1* (*P* = 0.019) and *Anoxybacillus* (*P* = 0.031) decreased over time (Fig. 4B). The other three MPG genera (*Dolosigranulum*, Staphylococcus, and Streptococcus) remained relatively stable during the follow-up period (*P* > 0.05 for all).

### Dynamic patterns of NPM are comparable between AE and AS patients.

**FIG 5:**
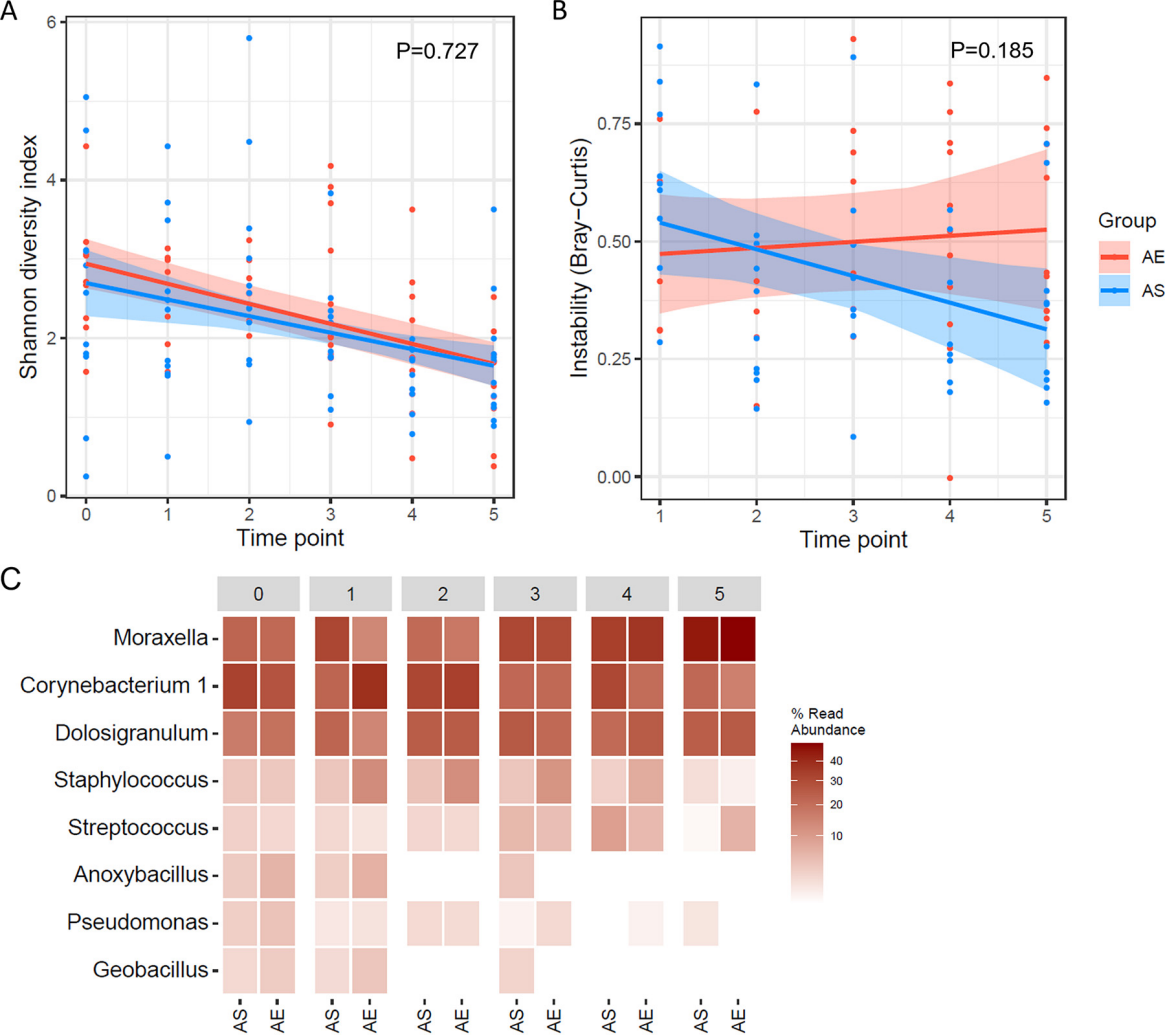
Dynamic changes in NPM diversity and composition over time among asthmatic children using LME modeling. (A and B) Alpha diversity based on Shannon diversity index (A), and beta diversity based on Bray Curtis distance (B) are plotted against time points. Red and blue lines are regression lines from LME models for AE and AS groups, respectively. Shading indicates the 95% confidence interval (CI). Points denote samples from AE (in red) and AS (in blue) groups. (C) Heatmap shows the average relative abundances of the top 8 most abundant genera by time point in AE and AS groups. AE, asthma exacerbation; AS, stable asthma. Time point 0 refers to baseline.

Twenty-four asthmatic patients who had ≥ 3 longitudinal FNPS samples (including baseline and scheduled visits) were included in the longitudinal analyses (*n* = 121, Fig. 1A). These patients were categorized into AE (N = 11) and AS (N = 13) groups, with the baseline clinical characteristics being comparable between the two groups (Wilcoxon rank-sum test or Fisher’s exact test, *P* > 0.05 for all; Table S1). We assessed the temporal variations of alpha and beta diversity as well as taxa abundances using LME models. In these models, time points, asthma groups, and their interactions were included as fixed effects and the subject as a random effect of both the intercept and the slope of time points. We found that NPM alpha diversity (SDI) significantly decreased (*P* < 0.001; Table 2) over time. However, the changing patterns were comparable (*P* = 0.727; Fig. 5A and Table 2) between AE and AS children. These observations were robust to other alpha diversity measures (Fig. S3A to C and Table 2). Regarding beta diversity, we did not detect a significant trend over time in Bray-Curtis distance between same-subject NPMs sampled in consecutive time points (*P* = 0.398; Table 2). However, although AE and AS groups exhibited a similar temporal pattern in beta diversity (Bray-Curtis distance, *P* = 0.185), we observed a trend toward increased dissimilarity between consecutive NPM in AE patients and an opposite trend in AS group (Fig. 5B), which was particularly so based on Jaccard distance (Fig. S3D, *P* = 0.023). Similar findings were also obtained using other beta diversity measures (Fig. S3D to F, Table 2), suggesting higher temporal stability of NPM composition in AS than AE patients. Furthermore, these changing patterns in the alpha and beta diversity of NPM held even when we considered nonexacerbated samples only (*n* = 116, Fig. S4).

**TABLE 2:** Linear mixed-effect (LME) model results for NPM alpha, beta diversity, and taxa abundances

Category	Response	Fixed effects		Interaction terms
		Time^*a*^	Asthma group	Time: asthma group
Alpha diversity	Shannon diversity index	**<0.001**	0.537	0.727
	Pielou’s evenness index	**<0.001**	0.577	0.419
	Observed ASVs	**<0.001**	0.670	0.431
	Faith’s phylogenetic diversity	**<0.001**	0.674	0.316
Beta diversity	Bray-Curtis distance	0.398	0.342	0.185
	Jaccard distance	0.403	0.665	**0.023**
	Unweighted UniFrac distance	0.363	0.614	0.144
	Weighted UniFrac distance	0.193	0.782	0.198
Relative abundances of the top eight genera	*Moraxella*	**0.006**	0.561	0.465
	*Corynebacterium 1*	**0.021**	0.602	0.311
	*Dolosigranulum*	0.106	0.593	0.420
	Staphylococcus	0.172	0.084	0.507
	Streptococcus	0.294	0.687	0.546
	*Anoxybacillus*	**0.035**	0.386	0.381
	Pseudomonas	**<0.001**	0.131	0.226
	*Geobacillus*	0.051	0.515	0.476

a LME modeling was performed using the nlme::lme function in R. Significance (*P* values) of LME model analyses was determined by ANOVA. Significant *P* values (<0.05) were indicated in bold.

We next examined temporal patterns of the top eight most abundant genera (mean relative abundance >1%) that accounted for 92.6% of quality-filtered sequences in the data set. Consistent with previous findings (Fig. 4B), *Moraxella* (*P* = 0.006) significantly increased while *Corynebacterium 1* (*P* = 0.021), *Anoxybacillus* (*P* = 0.035) and Pseudomonas (*P* < 0.001) decreased over time (Fig. 5C and Table 2). Nevertheless, longitudinal patterns in the abundances of all top eight genera were similar between AE and AS groups (LME models, *P* > 0.05 for all; Table 2). Dynamic changes in relative proportions of these eight genera were further visualized with volatility analysis (Fig. S5 to S7). Collectively, these findings implied that short-term temporal dynamics of NPM communities were not related to AE in children.

### NPM underwent *Moraxella* expansion during AE and showed remarkable resilience afterward.

**FIG 6:**
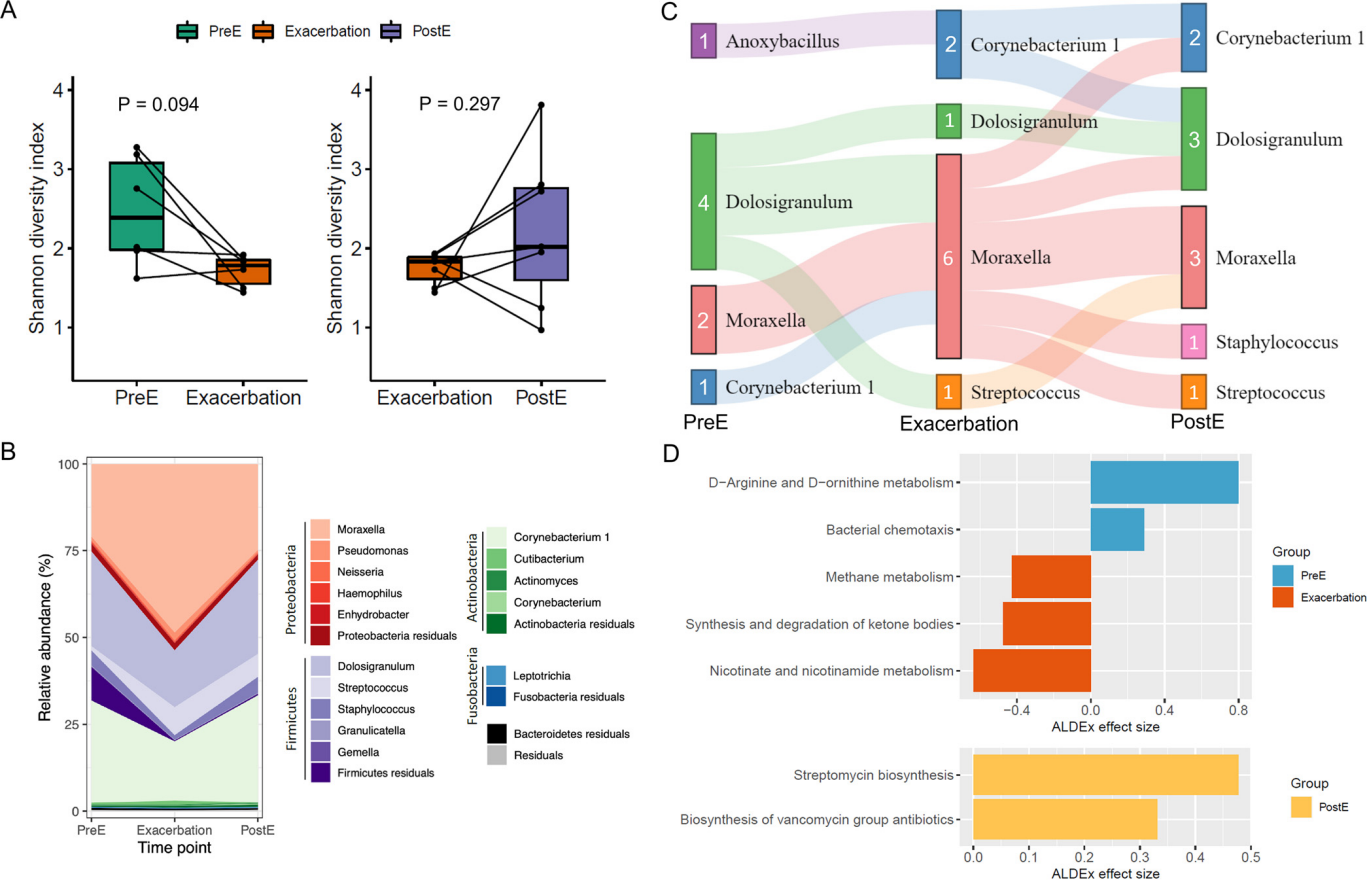
Taxonomical and functional changes in NPM at three phases of exacerbation. (A) SDI of nasopharyngeal microbial communities showed a trend toward decrease during exacerbation. Black lines connect samples from the same AE subject. Boxes represent the median, lower and upper quartiles, respectively. The ends of the whiskers indicate the minimum and maximum of the data after the removal of outliers. (B) Stacked area chart showing the relative abundances of the top 15 ASVs based on 90^th^ percentiles. ASVs were color-coded according to their phylum-level and genus-level taxonomic classification. ASVs from the five most abundant phyla were further stratified by genera, with other ASVs collapsed as “residuals”. (C) Sankey diagram indicating MPG transitions throughout the pre-exacerbation, during, and postexacerbation stages. Genera names representing MPGs were shown beside the colored boxes. Numbers denote the number of samples belonging to the respective MPGs. (D) KEGG pathways were significantly differentially abundant during exacerbation compared to PreE (upper panel) or PostE (bottom panel). PreE, pre-exacerbation; Exacerbation, during exacerbation; PostE, postexacerbation.

To investigate the temporal fluctuations of NPM during AE, we assessed changes in microbial diversity and taxa abundances for AE subjects over three phases of exacerbation (i.e., pre, during, and post). Twenty-eight samples from 10 AE patients were included and one AE patient with an NPM profile available at only one time point was excluded. Alpha diversity (SDI) was lower in exacerbation than pre-exacerbation (PreE, *P* = 0.094) and postexacerbation (PostE, *P* = 0.297, Wilcoxon signed-ranked test) samples (Fig. 6A). Intriguingly, the abundances of MPG genera experienced dramatic fluctuations during AE and exhibited extraordinary resilience after AE resolution. The mean relative abundance of *Moraxella* (48.5%) almost doubled at exacerbation compared to that in PreE (21%) and PostE (25%) samples. In contrast, *Corynebacterium 1* accounted for 17.1% of NPM at exacerbation, decreasing by more than 40% compared to both PreE (29.4%) and PostE (30.8%), respectively; *Dolosigranulum* also decreased by 39% at exacerbation (16.4%) compared to before and after exacerbation (27% for both PreE and PostE) (Fig. 6B). These fluctuations were further corroborated by MPG transition analyses. We observed that half of the samples (3/6) switched to *Moraxella* MPG during exacerbation from *Dolosigranulum*-dominated (two samples) or *Corynebacterium 1*-dominated (one sample) MPGs before exacerbation. These samples switched back to *Dolosigranulum*, *Corynebacterium 1,* or other MPGs after an exacerbation (Fig. 6C). These lines of evidence supported that *Moraxella* overgrowth played a pathogenic role in AE among schoolchildren.

### Altered metabolic pathways of NPM during AE were mainly contributed by *Moraxella*.

Taxonomic changes during exacerbation also corresponded to alternations in the functional potential of NPM communities. Through functional prediction of NPM on these 28 samples (PreE, Exacerbation, and PostE) using PICRUSt2, we identified a total of 168 KEGG pathways, among which 107 pathways (mean relative abundance >0.1%) were subjected to differential abundant test using ALDEx2. Eight pathways differed significantly (*P* < 0.05, Wilcoxon signed-rank test) in their community-total relative abundances across groups (Fig. 6D, Fig. S8, and Table S2). At a threshold effect size >0.3, abundances of d-arginine and d-ornithine metabolism (*P* = 0.013) was higher in the PreE group, while nicotinate and nicotinamide metabolism (*P* = 0.048), synthesis and degradation of ketone bodies (*P* = 0.045), and methane metabolism (*P* = 0.032) were more abundant in the exacerbation group. Biosynthesis of antibiotics and other secondary metabolites like streptomycin (*P* = 0.032) and vancomycin (*P* = 0.007) were enriched in postexacerbation samples (Fig. 6D and Table S2). Importantly, we observed a trend that the mean relative contribution of *Moraxella* to nicotinate and nicotinamide metabolism, commonly known as vitamin B3, has more than doubled at exacerbation (56.9 ± 32.5%) compared to both PreE (27.1 ± 37.2%) and PostE (23.2 ± 36.1%) groups (*P* = 0.094; Fig. S9 bottom). Similar trends were also observed for ketone bodies and methane metabolism (unpublished data). These findings echoed our previous result of doubled relative abundance of this genus at exacerbation (Fig. 6B), supporting that overrepresentation of *Moraxella* in the nasopharynx might provoke AE through modulating changes in microbial metabolism.

## DISCUSSION

In this prospective study, we comprehensively characterized the temporal dynamics of NPM associated with AE by triweekly sampling at six time points over the autumn-winter period among Hong Kong children. Alpha diversity decreased while microbial composition remained stable over time in these children. The longitudinal patterns of NPM diversity and composition did not differ significantly between AE and AS patients, which suggested that the short-term temporal dynamics of NPM in asthmatics might not predict pediatric AE. NPM exhibited transient *Moraxella* expansion during AE and remarkable resilience after its resolution. Microbial pathways implicated in the metabolisms of vitamin B3, ketone bodies, and methane were markedly heightened at exacerbation compared to nonexacerbated states; these enhanced functions were primarily contributed by *Moraxella*.

This study analyzed the microbiome in the upper airway of children which represented the main literature due to ethical concerns for performing bronchoscopy in this age group (18–20). Several pediatric studies indicated that a nasopharyngeal swab was a good surrogate for endotracheal aspirates or bronchoalveolar lavage to investigate the airway microbiome (21, 22). Exacerbated nasopharyngeal samples showed lower microbial diversity compared to baseline and control groups, which contradicted a previous study showing higher NPM diversity in adults with AE than healthy subjects (14). This discrepancy might be explained in part by the age difference, with this factor being associated with baseline NPM composition in our study and that also reported by McCauley et al. (16).

During the surveillance period, we observed a steady decrease over time in the richness and evenness of NPM. This was discrepant from previous work by Pérez-Losada et al. (23) that reported no difference in NPM alpha diversity based on two sequential samples collected 5.5 to 6.5 months apart. One possible reason is fewer time points (only two) and the longer sampling interval across different seasons in that study might fail to capture short-term changes in NPM. Seasonal changes in NPM have been reported in healthy young children (24). The more frequent (every 2 to 4 weeks) and a larger number of sampling time points (six) in this study allowed more accurate and reliable characterization of NPM dynamics in asthmatic children. In contrast, we and others (23) found that NPM composition did not change over time, suggesting temporal stability of NPM community structure in asthmatics. Interestingly, although the longitudinal patterns of both alpha and beta diversity were similar between AE and AS groups, we did observe a trend toward lower Bray-Curtis dissimilarity of NPM between consecutive time points in AS patients and an opposite trend among patients with AE. This might reflect higher stability in the upper airway microbiome among the former that reduced their susceptibility to AE.

The major genera in NPM showed differential longitudinal changes in their relative abundances during the surveillance period. *Moraxella* gradually increased over time and MPG dominated by this genus exhibited temporal stability, supporting a previous finding of *Moraxella* being a stable colonizer in the nasopharynx of asthmatic children (16). *Dolosigranulum*-dominated MPG also showed temporal stability in asthmatic children, which has not been reported before. Our observation that MPGs changed from being dominated by *Dolosigranulum or Corynebacterium 1* before exacerbation to *Moraxella* dominance at the time of AE was strikingly consistent with the finding from a prospective US cohort (15). This phenomenon might indicate an antagonistic relationship through competitive colonization between *Moraxella* and these two genera during AE. Indeed, antibacterial products produced by *Corynebacterium* and *Dolosigranulum* prevented the growth and nasal colonization of Streptococcus (25, 26), a pathogen associated with recurrent wheezing and childhood asthma (27, 28). It is worth noting that the role of the *Dolosigranulum* in asthma remains controversial. We previously found *Dolosigranulum* to be associated with a lower risk of wheezing illnesses in preschool children (13), whereas Kim et al. (29) reported a higher proportion of this genus in asthma and proposed that *Dolosigranulum* could increase the risk of respiratory tract infection. Further investigations are needed to elucidate mechanisms of nasopharyngeal cocolonization interactions among *Dolosigranulum*, *Corynebacterium 1*, and *Moraxella* in AE.

All the six identified MPGs except *Anoxybacillus* have been reported previously (16, 30). In Western asthmatic children, Haemophilus-MPG was more commonly identified from the nasopharynx (16, 20). *Anoxybacillus* was one of the most abundant airborne thermophilic bacterial genera in China (31) that contaminated processed food products, which were not initially considered human colonizers (32). However, a recent study reported that *Anoxybacillus* enrichment in the lower respiratory tract might suppress allergies in Chinese children (33). We revealed that this genus decreased with time independent of AE, suggesting *Anoxybacillus* to be a transient colonizer of NPM with a potentially protective role in Asian asthmatic children. This finding may reflect distinct NPM dynamics of asthmatic children across geographically different populations.

We found significantly enhanced functions related to microbial metabolism of vitamin B3, ketone bodies, and methane at AE. Kelly et al. (34) reported a higher level of nicotinamide pathway metabolites in the plasma of asthmatic children. Elevated expression of NADP oxidase 4 (NOX4) in asthmatics promoted aberrant contractility of airway smooth muscle (35). Additionally, increased synthesis and degradation of ketone bodies at exacerbation suggests that NPM might regulate its ketone body metabolism during AE. This finding has not been reported previously in asthmatic patients although a previous study suggested that the gut microbiome could contribute to the elevated serum level of ketone body β-hydroxybutyrate in a mouse model of allergic asthma (36). Furthermore, methane metabolism, as an important pathway related to cellular energy production, was altered in asthmatic airways of Asian children based on the metabolomics of exhaled breath condensate (37). Another metabolomics study suggested perturbation of this pathway in AE children after combination treatment of inhaled budesonide and salbutamol (38). The elevated microbial methane metabolism during AE in this study corroborated these findings from the perspective of metagenomics, suggesting that dysregulated energy metabolism derived from methanogenic bacteria in the nasopharynx might play a role in AE. Given a higher abundance of *Moraxella* during AE has been observed in our study and others (16, 39), we further revealed that *Moraxella*’s relative contribution to these above-mentioned increased pathways also doubled at AE compared to non-AE time points, which provided a functional explanation for *Moraxella* species pathogenesis in AE. On the other hand, weakened biosynthesis of antibiotics such as streptomycin and vancomycin at AE might foster airway pathogens sensitive to these antibiotics and thus aggravate asthma.

In summary, we demonstrated that asthmatic children presented a dynamic pattern of NPM with reduced diversity and stable composition independent of AE when viewed at a tri-weekly timescale over 4 months. Transient expansion of *Moraxella* with altered NPM metabolic pathways during AE provided a clue to the mechanisms underlying *Moraxella*-related AE risk. Future multiomics studies aiming at unveiling the pathogenicity mechanisms of NPM in AE occurrence could pave the way for new therapeutic and preventive strategies for asthma.

## MATERIALS AND METHODS

### Study design and sample collection.

Thirty-three school-age (6 to 17 years) children of Chinese ethnicity were recruited from the allergy clinic of Prince Wales Hospital, a university-affiliated teaching hospital in Hong Kong. These children suffered from physician-diagnosed asthma, and they were exacerbation-prone as defined by a history of at least one AE within the past 12 months. These asthmatic subjects were prospectively followed up for asthma control status at regular intervals to investigate the relationship between NPM dynamics and AE occurrence.

AE was defined by one of the following criteria: (i) regular use of ≥3 doses of short-acting β_2_-agonist daily for ≥2 days, (ii) prescription of short-course oral prednisolone, or (iii) asthma-related unscheduled physician visit, emergency room visit or hospitalization. All patients received standardized asthma treatment according to Global Initiative for Asthma guidelines. Exclusion criteria for subjects include (i) history of structural lung disease; (ii) coexisting primary or secondary immunodeficiency; (iii) unwillingness for serial follow-up; and (iv) non-Hong Kong residents. Demographics, personal and family history of allergic diseases, and environmental exposures were collected by Chinese questionnaire of International Study of Asthma and Allergy in Childhood (ISAAC) (40). Subjects’ atopic status to common aeroallergens was assessed by skin prick test (SPT), and HDM exposure was evaluated by measuring *Der p* 1 concentration in settled mattress dust samples (41) by enzyme-linked immunosorbent assay (Indoor Biotechnologies, Cardiff, UK).

Samples and clinical metadata obtained at recruitment were treated as the baseline. Asthmatic subjects were then followed from September to December 2017, the peak season of HRV infection in Hong Kong (42), to collect FNPSs (Copan FLOQSwab, Brescia, Italy) from both nostrils at five planned home visits with 2-week to 4-week intervals. Patients’ asthma control status was evaluated by asthma diary, asthma control test (ACT) or childhood-ACT (C-ACT, for subjects under 12 years old), spirometry (MIR, Italy), and exhaled nitric oxide levels (eNO; NIOX VERO, Circassia, USA). ACT or C-ACT score >19 was considered good asthma control (43). Subjects who experienced any AE episodes during the surveillance period were deemed as AE group, and those without exacerbation events were considered the stable asthma (AS) group. Exacerbation samples were collected within 2 days from the onset of worsened asthma symptoms. Extra illness visits were arranged when AE occurred during the intervals that were beyond 2 days from the scheduled visits. Additionally, 20 schoolchildren without asthma history ever were recruited from the community in the same district as controls, for whom one cross-sectional FNPS sample was collected at recruitment. All subjects were free from upper respiratory tract infection (URTI) for at least 2 weeks and not exposed to any antibiotics within 4 weeks before recruitment. FNPS samples were transported to the laboratory on ice and stored at –80°C until analyses.

### DNA extraction, amplification, and 16S rRNA gene sequencing.

Fifty microliters of each FNPS sample was aliquoted for HRV detection by RT-PCR. RNeasy Minikit (Qiagen) was used for viral RNA extraction and PrimeScript RT reagent kit (TaKaRa, Japan) for reverse transcription-PCR (RT-PCR) using HRV-specific primers. Total genomic DNA was extracted using MO BIO Power Soil DNA isolation kit (Qiagen) according to the manufacturer’s protocol for low biomass samples. Extracted DNA was quantified with a Qubit 4 Fluorometer, and samples with ≥50 ng DNA were retained for further processing. Library preparation was performed using GeneRead DNA Library I kit (Qiagen), and the V4 region of the bacterial 16S rRNA gene was amplified using the 515F/806R primer pair and purified as described previously (44). Quantified and pooled amplicons were sequenced on Illumina HiSeq 2500 platform (Illumina Inc., San Diego, CA, USA) to generate paired-end (2 × 250-bp) reads.

### Microbiome data analyses.

Demultiplexed reads from two separate sequencing runs were imported into QIIME2 (45) (version 2020.2) and subjected to quality control using DADA2 (46), respectively. DADA2 trimming parameters were: 19 to 230 bp forward and 20 to 220 bp reverse reads for run 1,19 to 209 bp forward and 20 to 136 bp reverse reads for run 2, which removed low-quality bases with Phred33 quality score <35. The filtered sequences were then subjected to a high-resolution sample inference process by the DADA2 algorithm to retrieve exact amplicon sequence variants (ASVs). ASV tables from the two runs were merged into one, and taxonomy assignment of the resulting ASVs was performed based on SILVA 132 reference database with 99% similarity. To minimize the impact of various sequencing depths, we rarefied the samples to an even sampling depth (22,201 reads) at which all samples were retained while all taxa present within a sample were captured (Fig. S1). The rarefied ASV table was used for all downstream analyses.

We used four metrics to indicate alpha diversity: SDI, Pielou’s evenness index, observed ASVs, and Faith’s phylogenetic diversity (Faith PD). Another four metrics were used to represent beta diversity: Jaccard distance, Bray-Curtis distance, unweighted UniFrac and weighted UniFrac distances. All these metrics were calculated using the QIIME2 q2-diversity plugin. Permutational multivariate ANOVA (PERMANOVA) was applied to assess the association of between-sample community dissimilarity (beta diversity) with a range of clinical variables using the adonis function in the vegan R package (1,000 permutations). Procrustes analysis of Bray-Curtis distance was performed in QIIME2 to compare the principal coordinate matrices between paired baseline and exacerbation samples, with the significance of the correlation being assessed by a mantel test. A PCoA plot was generated using the R package ‘ampvis2’ (47) to visualize dissimilarity between samples.

To analyze the NPM dynamics, we adopted a modified concept of microbiome profile groups (MPGs) (20, 30) to classify samples. In brief, samples were assigned to MPGs based on the most abundant (dominant) genus in each sample. The relative abundances of MPG genera in individual samples were subjected to hierarchical clustering based on Bray-Curtis distance and complete linkage implemented by *hclust* R function. Volatility analysis was performed using the ‘volatility’ action of the q2-longitudinal plugin to display dynamic changes in the topmost abundant genera.

Metagenome prediction of NPM was conducted on a subset of 28 samples from AE patients using Phylogenetic Investigation of Communities by Reconstruction of Unobserved States version 2 (PICRUSt2, v2.3.0-b) (48) to assess the metabolic alterations during AE compared with pre-AE and post-AE time points. Specifically, the rarefied ASV table from QIIME2 was filtered to remove extremely rare ASVs (present only in one sample or with a total frequency <2), resulting in 366 ASVs to which PICRUSt2 was applied with the --per_sequence_contrib option. The generated gene family abundances were employed to infer KEGG pathway-level information with MinPath (49). A weighted Nearest Sequence Taxon Index (NSTI) score was calculated to assess the accuracy of PICRUSt2 predictions for each sample, which measured the similarity between bacteria from a given sample and the reference genome sequences. Five ASVs with an NSTI score >2 were excluded from downstream analysis (50).

### Statistical analysis.

All statistical analyses were performed using R version 4.0.3 (51) unless stated otherwise. The .qza files derived from QIIME2 were imported into R using qiime2R (52). Categorical and continuous variables were compared between groups using the Chi-square test and Wilcoxon rank-sum test, respectively. Alpha diversity across three or more groups was compared by the Kruskal-Wallis test followed by Dunn’s *post hoc* test. LME models (50, 53, 54) were performed using the nlme::lme function in R to determine the longitudinal changes in alpha and beta diversity as well as taxa abundances. We modeled time points, asthma group (AE versus AS), and their interaction as fixed effects and used subjects as a grouping variable in the random effect of time. Partial residual plots were generated using the R package ‘visreg’ (55) to represent effects.

NPM transitions were evaluated by analyzing MPG stability between consecutive samples from the same subjects (30). Specifically, transition into the same MPG between two consecutive time points (T1, T2) was considered a stable transition. The observed frequencies were calculated as the proportion of stable transitions among all observed transitions, with 95% confidence intervals (CI) estimated from 1,000 bootstrapping. The expected frequency of a particular MPG was defined as the square of the proportion of samples belonging to that MPG at T1 under the assumption of constant frequencies and random transitions. An MPG was considered significantly more stable than expected if the expected frequency was less than the lower limits of 95% CI of the observed frequency of stable transition, and less stable if the expected value was greater than the upper limits.

For metagenome inference, pathway data were normalized by centered log-ratio transformation (CLR). The R package ‘ALDEx2’ (version 1.22.0) (56) was used to identify significantly altered KEGG pathways based on Wilcoxon signed-rank test and Welch’s *t* test. A web tool BURRITO (57) was employed to visualize the contributing taxa for significant pathways. Statistical significance was set as a *P* < 0.05. *P* values were adjusted for multiple testing by Benjamini-Hochberg false discovery rate (FDR) method.

### Ethics approval and consent to participate.

The project was approved by the Joint Chinese University of Hong Kong-New Territories East Cluster Clinical Research Ethics Committee (reference no. 2017.031). All participants provided written informed consent.

### Data availability.

Sequences were available in the NCBI Sequence Read Archive with the accession number PRJNA748666. In-house R scripts used to generate figures were available on GitHub (https://github.com/Jessie-HOU/microbiome/tree/master).
