# Supplementary material for: Temporal Dynamics of the Nasopharyngeal Microbiome and its Relationship with Childhood Asthma Exacerbation
Source: Microbiol Spectr. 2022 May 12;10(3):e00129-22. doi: 10.1128/spectrum.00129-22 (PMC9241764; doi:10.1128/spectrum.00129-22)
Supplement: SUPPLEMENTAL FILE 1 — Supplemental material. Download spectrum.00129-22-s001.pdf, PDF file, 1.7 MB [file spectrum.00129-22-s001.pdf]

Online Supplement

Temporal dynamics of the nasopharyngeal microbiome and its relationship with childhood asthma exacerbation

Jinpao Hou,[a,e]* Yuping Song,[b]* Agnes Sze Yin Leung,[b] Man Fung Tang,[b,d] Mai Shi,[a,e] Evy Yiwei Wang,[a,e] Joseph Gar Shun Tsun,[b] Renee Wan Yi Chan,[b,c,d] Gary Wing Kin Wong,[b] Stephen Kwok-Wing Tsui,[a,e,f] § Ting Fan Leung[b,c,d§]

[a]School of Biomedical Sciences, The Chinese University of Hong Kong, Hong Kong

[b]Department of Paediatrics, The Chinese University of Hong Kong, Prince of Wales Hospital, Hong Kong

[c]Chinese University of Hong Kong-University Medical Center Utrecht Joint Research Laboratory of Respiratory Virus and Immunobiology, The Chinese University of Hong Kong, Hong Kong

[d]Hong Kong Hub of Paediatric Excellence, The Chinese University of Hong Kong, Hong Kong

[e]Hong Kong Bioinformatics Centre, The Chinese University of Hong Kong, Hong Kong

[f]Centre for Microbial Genomics and Proteomics, The Chinese University of Hong Kong, Hong Kong

* These authors contributed equally as co-first authors.

§ Co-corresponding authors.

Supplemental Tables

Table S1 Baseline characteristics of the asthmatic children

	**Asthma exacerbation (AE, n=11)**	**Stable asthma (AS, n=13)**	***P-value****
Age (SD)	11.09 (3.02)	12.62 (2.81)	0.214
Gender (Male %)	8 (72.7)	6 (46.2)	0.240
Controlled asthma (%)	7 (72.7)	12 (76.9)	1.000
Regular ICS (%)	6 (54.5)	2 (15.4)	0.082
BMI (SD)	12.64 (7.42)	16.85 (9.25)	0.238
HRV infection	3 (27.3)	1 (7.69)	0.300
HDM allergy (%)	72.7	100	0.082
Der p 1 level in mattress dust (mg/g) (SD)	0.46 (0.44)	0.69 (1.29)	0.571
HDM allergy and exposure (%)	2 (18.2)	2 (15.4)	1.000
Siblings (%)	9 (81.8)	10 (76.9)	1.000
Pet (%)	1 (9.09)	3 (23.1))	0.596
Smoke exposure (%)	4 (36.4)	1 (7.69)	0.142
Baseline lung function			
FEV_1 (%Pred)	91.6 (22.2)	90.8 (13.5)	0.907
FEV_1/FVC (%)	84.8 (9.22)	91.0 (6.24)	0.063
PEF (%Pred)	80.6 (21.0)	82.46 (17.6)	0.810
FeNO (ppb)	33.4 (23.7)	43.92 (30.2)	0.358

Categorical variables were shown in counts with percentage in the parentheses; continuous variables were shown in mean with standard deviation in the parentheses. *, P-values were

calculated based on Fisher's exact test and Wilcoxon rank sum test as appropriate. Abbreviations: BMI, body mass index; N, number of patients; HRV, human rhinovirus; ICS, inhaled corticosteroid; HDM, house dust mites; FEV_1, forced expiratory volume during the first second of forced expiration; FVC, forced vital capacity; PEF, peak expiratory flow; FeNO, fractional exhaled nitric oxide level.

Table S2 Summary of significantly differential KEGG pathways by ALDEx2 analysis

KEGG pathway	we.ep	we.eBH	wi.ep	wi.eBH	Effect size	Comparison	Enrichment Group
**D Arginine and D ornithine metabolism**	0.010	0.501	0.013	0.436	0.799	PreE vs. Exac	PreE
**Dioxin degradation**	0.043	0.656	0.045	0.936	-0.795	PostE vs. PreE	PreE
**D Arginine and D ornithine metabolism**	0.018	0.656	0.038	0.936	-0.689	PostE vs. PreE	PreE
**Nicotinate and nicotinamide metabolism**	0.045	0.523	0.048	0.452	-0.636	PreE vs. Exac	Exacerbation
**Streptomycin biosynthesis**	0.038	0.990	0.032	0.886	0.477	PostE vs. Exac	PostE
**Synthesis and degradation of ketone bodies**	0.026	0.523	0.045	0.450	-0.473	PreE vs. Exac	Exacerbation
**Methane metabolism**	0.046	0.524	0.032	0.447	-0.425	PreE vs. Exac	Exacerbation
**Biosynthesis of vancomycin group antibiotics**	0.032	0.991	0.007	0.706	0.331	PostE vs. Exac	PostE
**Bacterial chemotaxis**	0.013	0.501	0.013	0.435	0.289	PreE vs. Exac	PreE

Pairwise comparisons of KEGG pathways by ALDEX2 identified eight pathways that are significantly ($P<0.05$) differentially abundant between groups based on both Wilcoxon signed rank test and Welch’s t-test, which are listed in the table above ordered by effect size. Abbreviations: we.ep, P-value of Welch’s t-test; we.eBH, Benjamini-Hochberg (BH) adjusted P-value of Welch’s t-test; wi.ep, P-value of Wilcoxon signed rank test; wi.eBH, BH adjusted P-value of Wilcoxon signed rank test.

Supplementary Figures

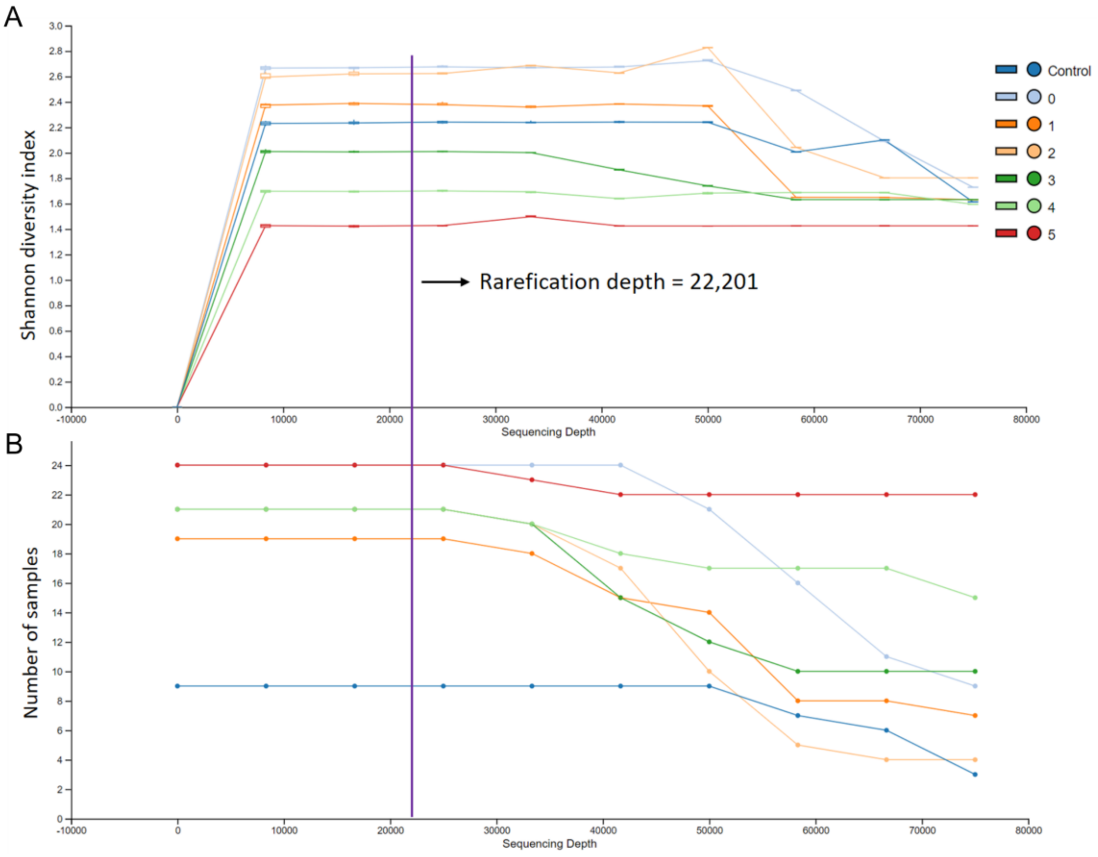

Fig. S1 Rarefaction curves of nasopharyngeal samples from all samples. A, Rarefaction curves of the alpha diversity at each of the time point (i.e. visit) levels off as the sequencing depth increased. **B,** Remaining samples at each rarefaction step. The purple vertical line indicates the position of rarefication depth. Control means non-asthma samples; Time point 0 refers to baseline, while 1, 2, 3, 4, 5 denote the subsequent sampling visits. The rarefication curve of Shannon diversity index (SDI) showed the maximum microbial diversity reached at the sequencing depth of 10,000-25,000, where meanwhile all samples were retained.

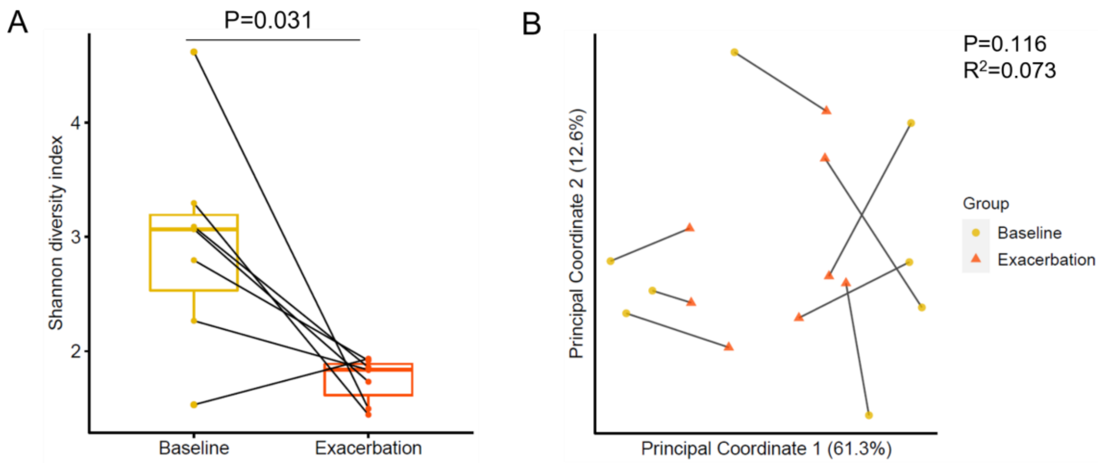

Fig. S2 (related to Fig. 3) Differences in NPM alpha and beta diversity between paired baseline and exacerbated samples from the same asthma patient. A, Shannon diversity is lower in exacerbation samples than baseline samples. Wilcoxon sign-ranked test was performed with one outlier sample pair excluded. **B,** PCoA plots from Procrustes analysis of Bray-Curtis distances in pairwise samples. Baseline samples in yellow circle and their paired exacerbation samples in red triangle were connected by a line. A longer distance between paired samples indicates a lower degree of similarity in their NPM composition.

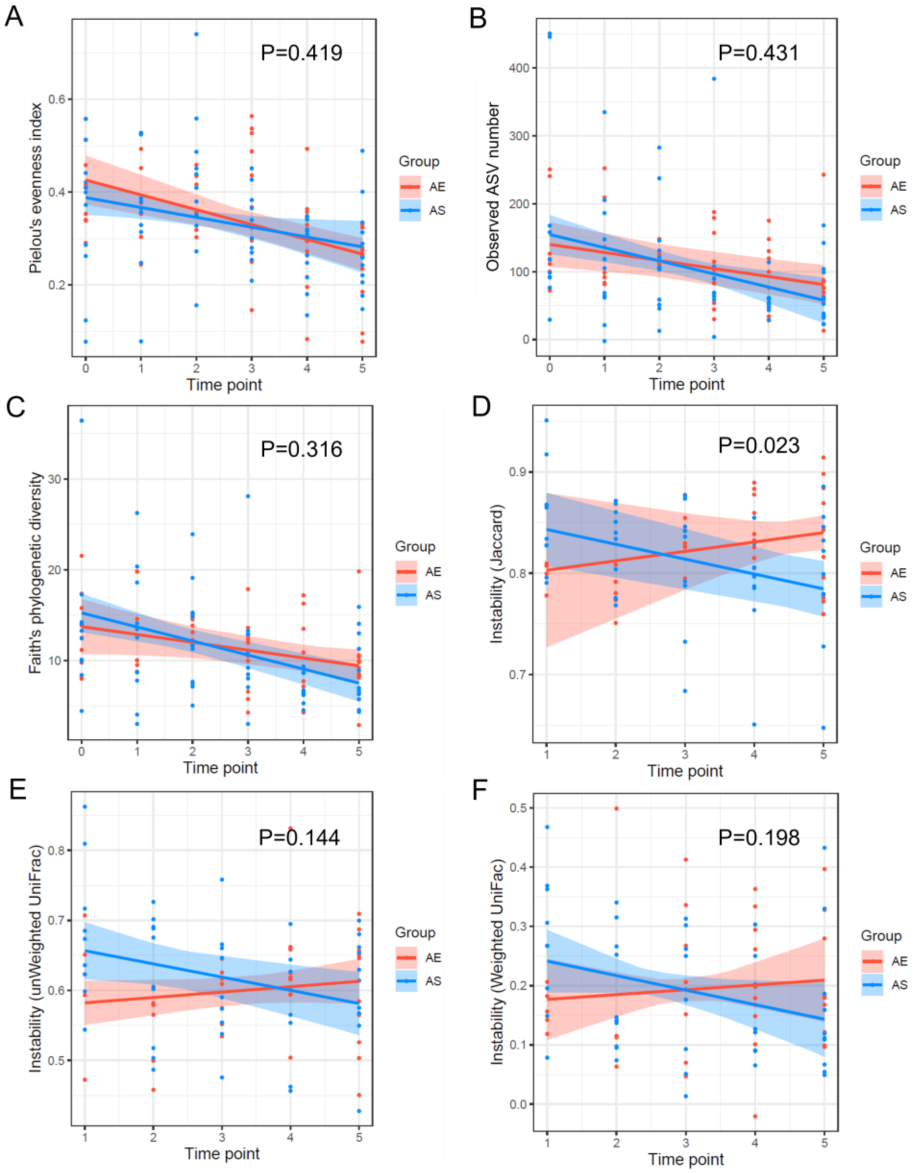

Fig. S3 (related to Fig. 5) Dynamic changes in NPM diversity over time among asthmatic children using LME modeling. Alpha diversity based on Pielou's evenness index **(A),** Observed ASVs **(B)** and Faith's phylogenetic diversity **(C)**, beta diversity based on Jaccard distance **(D)**, unweighted UniFrac distance **(E)** unweighted UniFrac distances **(F)** were plotted against timepoint. P-values indicate significances between groups. Red and blue lines are regression lines from LME models for AE and AS groups, respectively. Shading indicates the 95% confidence interval (CI). Points denote individual samples.

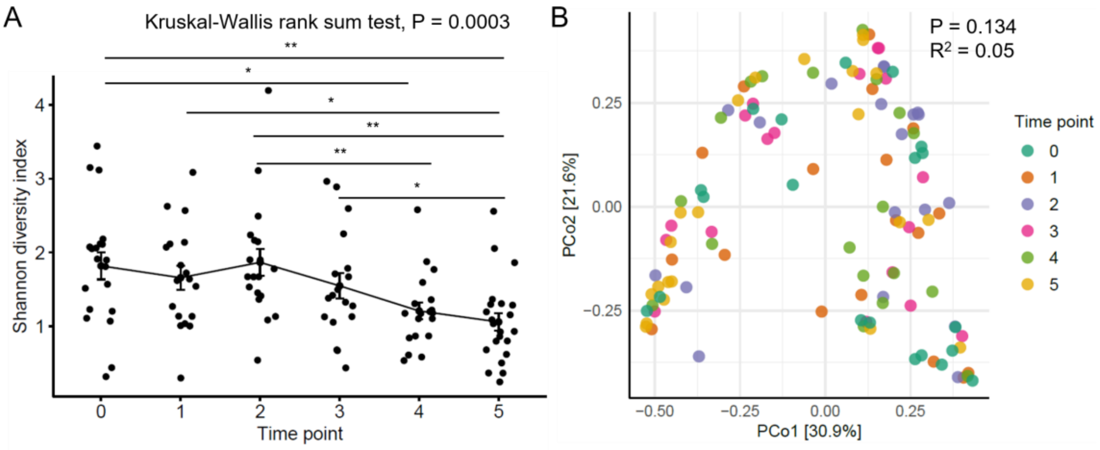

Fig. S4 Temporal changes in NPM diversity and composition among 116 non-exacerbated asthma samples. A, Shannon diversity of NP microbiome decreased over time. Kruskal-Wallis rank sum test and pairwise Wilcoxon test were used with Benjamini-Hochberg FDR correction. *, P<0.05; **, P<0.01. **B,** Principal coordinate analysis (PCoA) plots based on Bray-Curtis distances showed no distinct clusters among samples at different time points. PERMANOVA test was performed using Adonis2 function in vegan R package with 1,000 permutations.

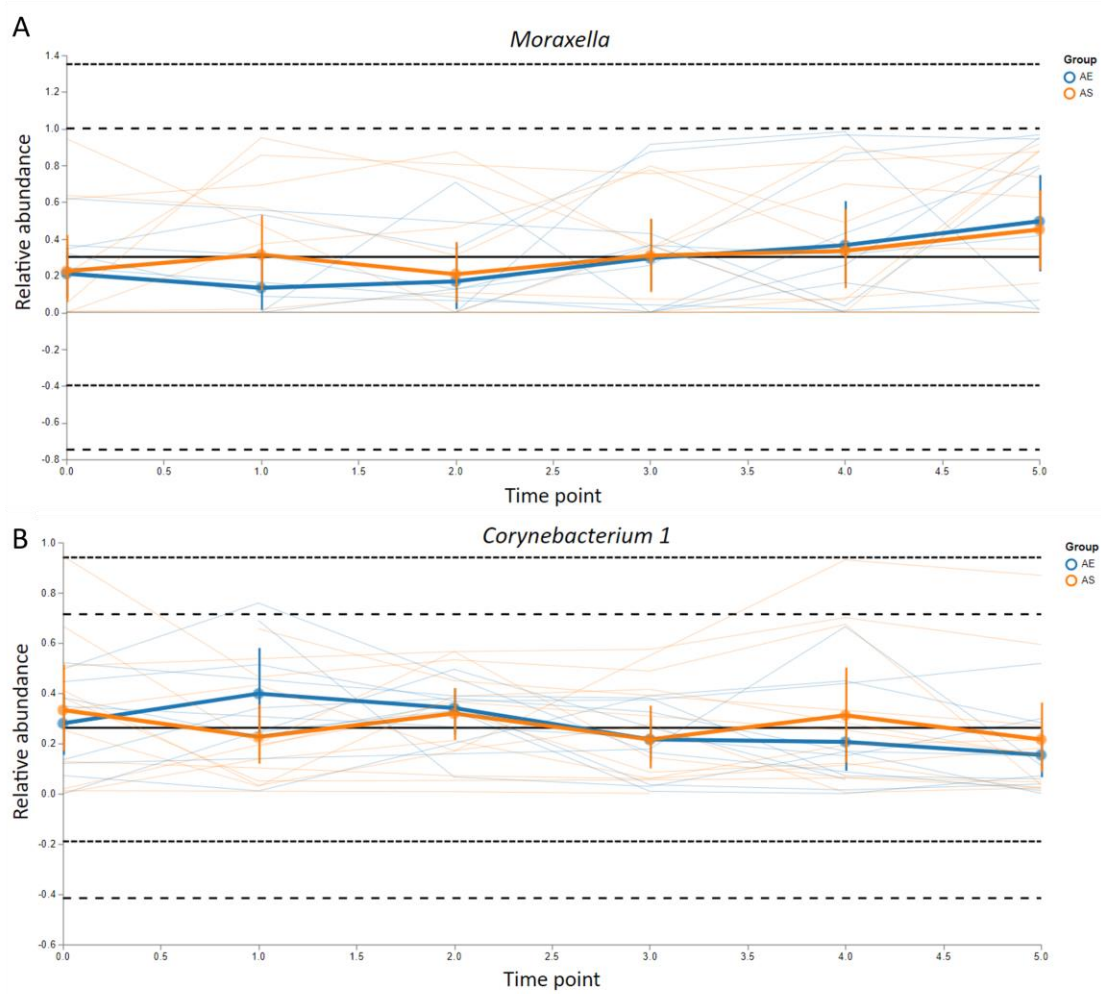

Fig. S5 (related to Fig. 5 and Table 2) Volatility analysis of the dynamic changes over time in relative abundances of the topmost abundant genera using LME modelling. **A,** *Moraxella*. **B,** *Corynebacterium 1*. The bold lines denote mean proportion for group, while the thin lines ('spaghetti') indicate the relative proportion of individual subjects. AE, asthma exacerbation; AS, stable asthma.

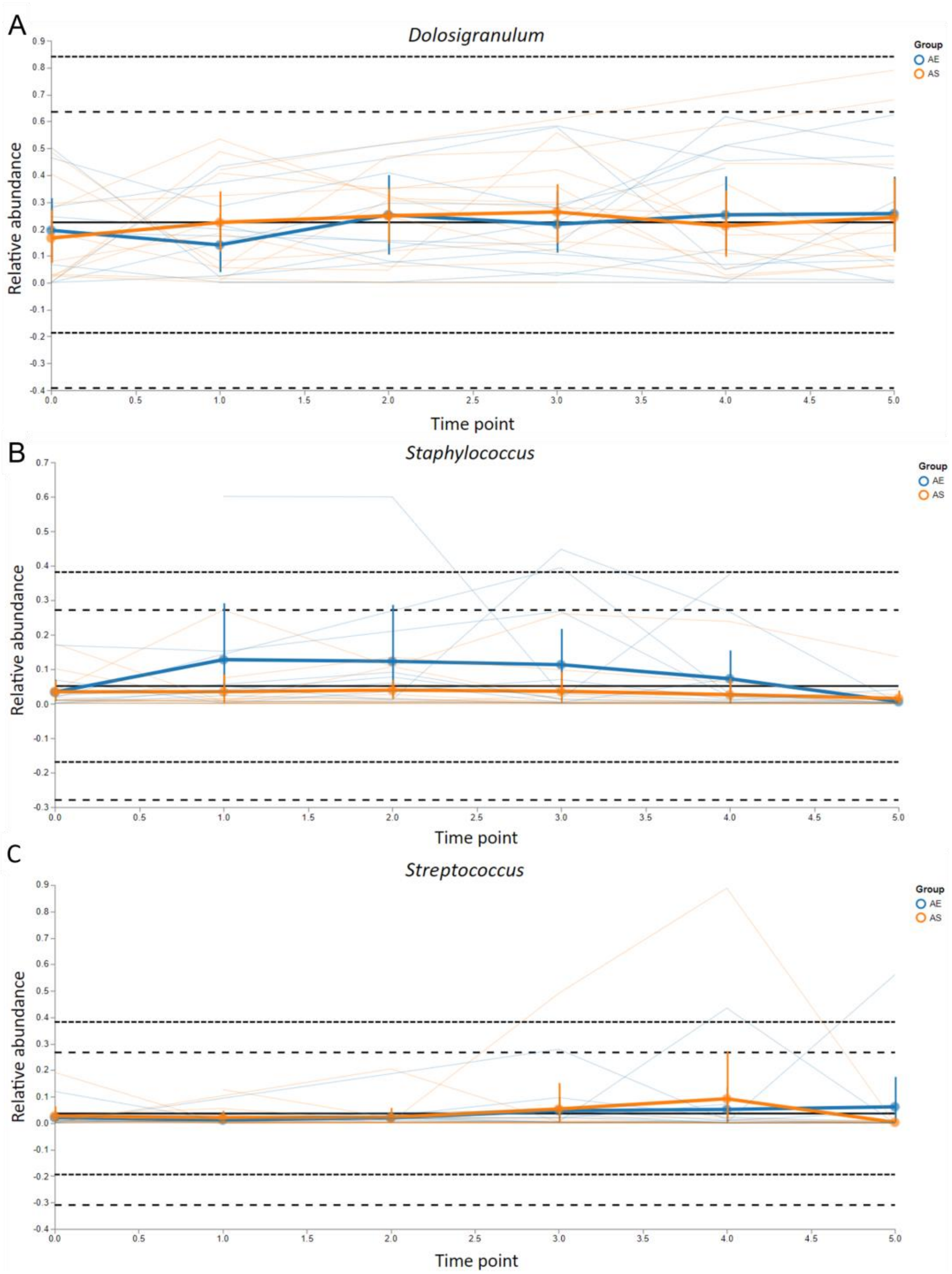

Fig. S6 (related to Fig. 5 and Table 2) Volatility analysis of the dynamic changes over time in relative abundances of the topmost abundant genera using LME modelling. **A,** *Dolosigranulum*. **B,** *Staphylococcus*. **C,** *Streptococcus*.

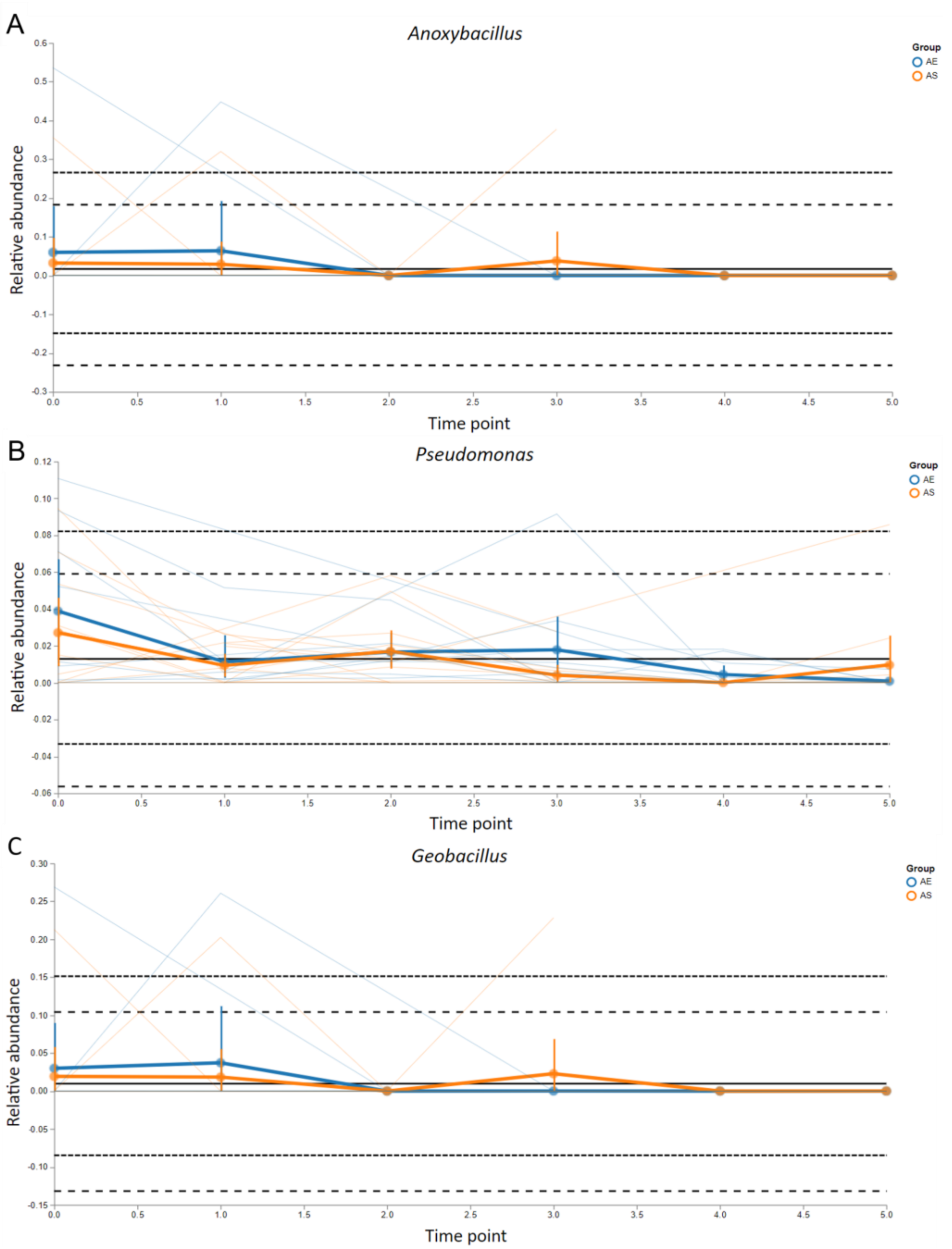

Fig. S7 (related to Fig. 5 and Table 2) Volatility analysis of the dynamic changes over time in relative abundances of the topmost abundant genera using LME modelling. **A,** *Anoxybacillus*. **B,** *Pseudomonas*. **C,** *Geobacillus*.

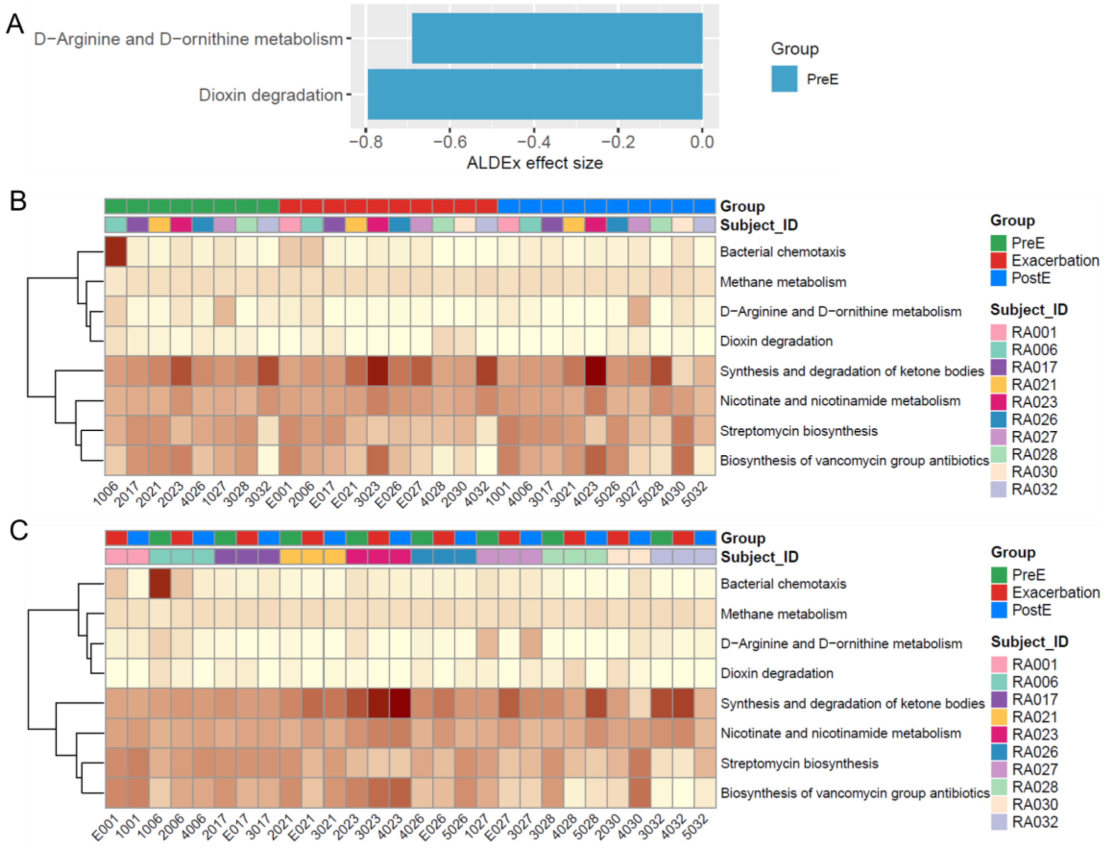

Fig. S8 (related to Fig. 6D) Metagenome prediction on NPM during asthma exacerbation. A, Significantly differentially abundant KEGG pathways between PreE and PostE samples identified by ALDEx2. Comparisons between Exacerbation and non-exacerbated stages (i.e., PreE and PostE) were shown in Fig. 6D. **B-C**, Heatmap showing the community-total relative abundances of the eight altered KEGG pathways in individual samples among 10 AE subjects. These pathways were clustered based on Euclidean distance. Samples were ordered by Group (B) and Subject ID (C), respectively. PreE, pre-exacerbation; Exacerbation, during exacerbation; PostE, post-exacerbation.

Fig. S9 (The figure legend is shown in the next page)

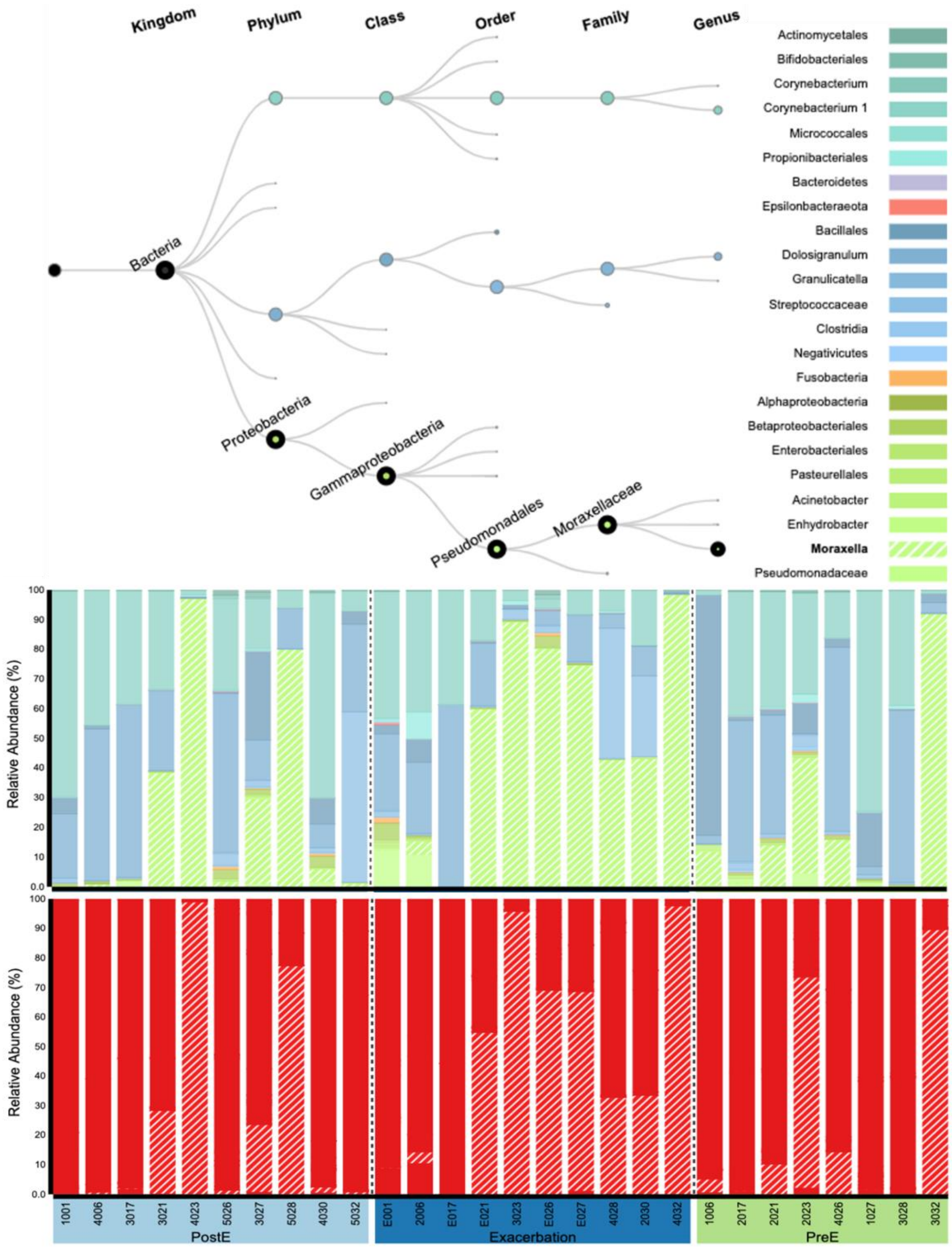

Fig. S9 Relative abundances of contributing taxa for Nicotinate and nicotinamide metabolism visualized with BURRITO. This function was primarily contributed by *Moraxella*, *Corynebacterium 1* and *Dolosigranulum* that showed the largest node size in the upper panel. The relative taxonomical and contributional abundances of these taxa in individual samples were shown in the middle and bottom panels of the plot, respectively, on which the diagonal stripes represented Moraxella. PreE, pre-exacerbation; Exacerbation, during exacerbation; PostE, post-exacerbation.
